# A *Gossypium* BAC clone contains key repeat components distinguishing sub-genome of allotetraploidy cottons

**DOI:** 10.1186/s13039-016-0235-y

**Published:** 2016-03-22

**Authors:** Yuling Liu, Renhai Peng, Fang Liu, Xingxing Wang, Xinglei Cui, Zhongli Zhou, Chunying Wang, Xiaoyan Cai, Yuhong Wang, Zhongxu Lin, Kunbo Wang

**Affiliations:** State Key Laboratory of Cotton Biology, Institute of Cotton Research of Chinese Academy of Agricultural Science, Anyang, Henan 455000 China; National Key Laboratory of Crop Genetic Improvement, Huazhong Agricultural University, Wuhan, 430070 China; Anyang Institute of Technology, Anyang, Henan 455000 China

**Keywords:** *Gossypium*, BAC, Repetitive sequences, LTR-RT, FISH

## Abstract

**Background:**

Dissecting genome organization is indispensable for further functional and applied studies. As genome sequences data shown, cotton genomes contain more than 60 % repetitive sequences, so study on repetitive sequences composition, structure, and distribution is the key step to dissect cotton genome.

**Results:**

In this study, a bacterial artificial chromosome (BAC) clone enriched in repetitive sequences, was discovered initiatively by fluorescence in situ hybridization (FISH). FISHing with allotetraploidy cotton as target DNA, dispersed signals on most regions of all A sub-genome chromosomes, and only middle regions of all D sub-genome chromosomes were detected. Further FISHing with other cotton species bearing A or D genome as target DNA, specific signals were viewed. After BAC sequencing and bioinformational analysis, 129 repeat elements, size about 57,172 bp were found, accounting for more than 62 % of the BAC sequence (91,238 bp). Among them, a type of long terminal repeat-retrotransposon (LTR-RT), LTR/Gypsy was the key element causing the specific FISH results. Using the fragments of BAC matching with the identified Gypsy-like LTR as probes, the BAC-57I23-like FISH signals were reappeared. Running BLASTN, the fragments had good match with all chromosomes of *G. arboreum* (A_2_) genome and A sub-genome of *G. hirsutum* (AD_1_), and had relatively inferior match with all chromosomes of D sub-genome of AD_1_, but had little match with the chromosomes of *G. raimondii* (D_5_) genome, which was consistent with the FISH results.

**Conclusion:**

A repeats-enriched cytogenetic marker to identify A and D sub-genomes of *Gossypium* was discovered by FISH. Combined sequences analysis with FISH verification, the assembly quality of repetitive sequences in the allotetraploidy cotton draft genome was assessed, and better chromosome belonging was verified. We also found the genomic distribution of the identified Gypsy-LTR-RT was similar to the distribution of heterochromatin. The expansion of this type of Gypsy-LTR-RT in heterochromatic regions may be one of the major reasons for the size gap between A and D genome. The findings showed here will help to understand the composition, structure, and evolution of cotton genome, and contribute to the further perfection of the draft genomes of cotton.

## Background

*Gossypium*, as one of the best-characterized allopolyploid species, is divided into eight diploid genome groups (2n = 2× = 26), namely A-G and K, and one allotetraploidy genome group (2n = 4× = 52), which is allotetraploid bearing A and D genomes [1, 2]. So far, approximately 45 diploid and 6 tetrapolyploid *Gossypium* species are recognized [3, 4]. Among them, four cultivated species, the New World allopolyploids *G. hirsutum* and *G. barbadense* (2n = 4× = 52), and the Old World diploids *G. arboreum* and *G. herbaceum* (2n = 2× = 26), especially *G. hirsutum*, dominate worldwide cotton production. For a long time, cotton has been firmly established as the world’s most important fiber crop and an important source of seed oil and protein meal [5].

The two progenitors of allotetraploidy cotton diverged 4–8 million years ago, and re-hybridized about 1–2 million years ago [6, 7]. There is enough time for sequence divergence, as well as subsequent genome stability. What’s more, there is a wide range in genome size across closely related diploid species (from 880 Mb to 2572 Mb per haploid nucleus) and well-established phylogeny in *Gossypium* [8]. So, cotton is also an excellent model system for studying polyploidization, genomic organization, and genome-size variation. To dissect the genomic complexity in allotetraploidy cotton, extensive efforts have been performed. The ployploid parentage had been explained with the help of series of cytogenetic data combined with the observation derived from different studies. In early years, based on some classic cytogenetic and cytological studies, genome composition of the polyploids was investigated, which confirmed that the American allotetraploidy species are allopolyploids containing two resident genomes, an A-genome from Africa or Asia, and a D-genome similar to those found in the American diploids [9–11]. With the extensive application of FISH, more evidences that allotetraploidy cottons may be polyphyly have been obtained [12, 13].

It is believed that the proportion of protein-coding sequences is generally similar in different plant species [14], and repetitive DNA sequences are important factors in genome size variation [15–17]. Repetitive sequences can be classified into two categories: tandem repeats and transposable elements [18]. The former, which is usually found in specific genomic regions, such as centromeres or telomeres, has been extensively studied in different plant species [19–24]. Among the latter, retrotransposons replicating through a ‘copy and paste’ mechanism can result in the increase of the genome size to a great extent. Different methods had been used for analysis of repetitive DNA sequences, such as the low C_0_t analysis [25, 26], bacterial artificial chromosome (BAC) end sequences analysis [27], full-length BAC sequences analysis [28, 29]. To date, the most powerful method to characterize the high copy fraction of a genome is next generation sequencing and subsequent bioinformatic analysis [30, 31]. Recently, the draft assemblies of cotton genomes have been reported. More than 60 % of repetitive DNA sequences in genomes were revealed [32–36]. So dissecting the repetitive DNA sequences of genome is helpful to further understand the composition, evolution, and function of the cotton genome.

Fluorescence in situ hybridization (FISH), which allows direct mapping of DNA sequences on chromosomes, has become the most important technique in plant molecular cytogenetics [37]. Unique distribution patterns of repetitive DNA sequences on chromosomes has been revealed by FISH [38, 39], which provided a wealth of information regarding the chromosomal location of repetitive DNA sequences and their evolution in polyploidy genomes.

Here we analyzed a repeats-rich BAC clone combining FISH verification with sequence analysis, and identified the key elements resulting in specific FISH signal patterns, that is, a type of long terminal repeat-retrotransposon (LTR-RT). Simultaneous FISH with different cotton species as target chromosomes provided visual cytogenetic evidences of the colonization and size variation of the genomes. Moreover, by integrating FISH results with the cotton draft genomes, we preliminarily assessed the assembly quality of the draft genome assemblies.

## Methods

### Plant materials and BAC library

The cultivated *Gossypium* species, *G. hirsutum* (AD_1_) (accession TM-1), *G. barbadense* (AD_2_) (cultivar Hai-7124), and *G. arboreum* (A_2_) (cultivar Shixiya-1) were planted at Institute of Cotton Research of Chinese Academy of Agricultural Sciences (CRI-CAAS) in Anyang City, Henan Province, China. The wild species *G. tomentosum* (AD_3_) (accession P0601211), *G. mustelinum* (AD_4_) (accession P0811704), *G. darwinii* (AD_5_) (accession AD_5_-7), *G. raimondii* (D_5_) (accession D_5_-2), and the artificial hexaploid cotton (*G. hirsutum* (AD_1_) x *G. stocksii* (E_1_)) are perennially growing in National Wild Cotton Nursery in Sanya city, Hainan Island, China. The BAC library of *G. herbaceum* var. *africanum* was constructed by Gao et al. [40].

### BAC clone screening

During the screening of the 1^th^ chromosome-specific BACs from the BAC library of *G. herbaceum* var. *africanum*, with SSR markers derived from a whole-genome marker map [41], the BAC clone 57I23 enriched in repeats was found. The corresponding SSR marker Gh216, with primers (F/R): TCCACATTCCCATGCACTACTC/CTAAAACCTTATACATACAAAATGCAGC was used to screen the BAC library according to Cheng et al. [42] with a few modifications.

### BAC sequencing and repeats identification

The screened BAC clone 57I23 was sequenced and assembled by Shanghai Invitrogen Inc. Then BLASTN searches were performed using the BAC sequence as query, the draft genomes of cotton [33, 34, 36] as subjects respectively to detect the high copy repeats consisted in the BAC sequence. To further identify repeats types, online programs CENSOR (http://www.girinst.org/) [43], LTR-FINDER (http://tlife.fudan.edu.cn/ltr_finder/) [44] were used with the default parameters.

### Isolation of repeats

The primers of the selected repeats, with better match to genome or higher score in CENSOR results, were designed using NCBI primer-BLAST (http://www.ncbi.nlm.nih.gov/tools/primer-blast/). Touchdown PCR was performed to obtain amplification products with the BAC-57I23 bacterium as template. The amplification procedure was as follows: firstly, 98 °C 5 min for pre-degeneration; then 98 °C for 11 s, 52 + 1 °C for 18 s, 68 °C for 2.5 min for 10 cycles; 98 °C for 11 s, 57 °C for 18 s, 68 °C for 2.5 min for 30 cycles with a final extension at 68 °C for 6 min.

### DNA probes preparation

To visualize the distribution of the BAC-57I23 and its repeat elements, FISH was performed using BAC-DNA and repeat elements as probes respectively. BAC-DNA was isolated using Plasmid Miniprep Kit (Biomiga) according to the handbook. The PCR products were purified using Wizard SV Gel and PCR Clean-up System (Promega). They were labeled with DIG-nick translation Mix, according to the instructions of the manufacturer (Roche, USA).

### Chromosome preparation and FISH

Chromosome Preparation and the FISH procedure were conducted according to the previous protocols [45, 46]. The probes were detected with anti-digoxigenin-rhodamine (red) (Roche, USA). Images were captured using a CCD camera attached to a Zeiss Imager M1 microscope. Images were processed using Photoshop CS3.

## Results

### Discovery of the repeat-rich BAC clone 57I23

**Fig. 1:**
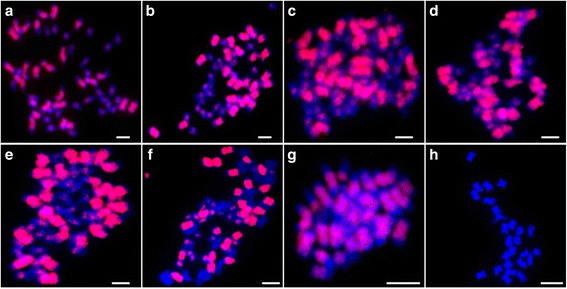
FISH mapping of BAC clone 57I23 on metaphase chromosomes of different *Gossypium* species. **a**-**h**: *G. hirsutum* (AD_1_, 2n = 4× = 52)*, G. barbadense* (AD_2_, 2n = 4× = 52)*, G. tomentosum* (AD_3_, 2n = 4× = 52)*, G. mustelinum* (AD_4_, 2n = 4× = 52)*, G. darwinii* (AD_5_, 2n = 4× = 52)*,* hexaploid hybrid (*G. hirsutum* × *G. stocksii*) (AADDEE, 3n = 6× = 78), *G. arboreum* (A_2_, 2n = 2× = 26)*, G. raimondii* (D_5_, 2n = 2× = 26). *Red*: the signal of BAC-57I23. Bar = 5 μm

During the screening of the 1^th^ chromosome-specific BACs from the BAC library of *G. herbaceum* var. *africanum*, a genome-specific BAC clone 57I23 was obtained using SSR marker Gh216, which was genetically mapped to AD_chr.01 (A_t_01) [47, 48]. FISHing with AD genome species as target DNA, the signals dispersed on the all chromosomes except the terminal areas of A sub-genome, and only middle areas of all D sub-genome chromosomes (Fig. 1a-e). So the FISH with BAC-57I23 can distinguish A sub-genome from D sub-genome simultaneously. Further FISHing with diploid A and D species, high coverage signals on all chromosomes of A genome were found (Fig. 1g), but hardly any signal on chromosomes of D genome (Fig. 1h). When using the artificial hexaploid hybrid (*G. hirsutum* x *G. stocksii*) preparation as target chromosomes, the similar A and D sub-genome signal patterns were observed, and none signal on E sub-genome (Fig. 1f). More than 15 metaphase cells with clear chromosome spreads were chosen to analyze the distribution of the FISH signals along the chromosomes. Based on the signal pattern, we deduced that the BAC clone 57I23 enriched in some types of repetitive elements.

### BAC sequencing and BLASTN analysis

To further understand the composition of BAC-57I23, BAC sequencing was performed by Shanghai Invitrogen Inc. Due to the existence of enriched repetitive sequences, three scaffolds with size of scaffold1-42,338 bp, scaffold2-26,803 bp, scaffold3-22,097 bp were obtained, respectively.

**Fig. 2:**
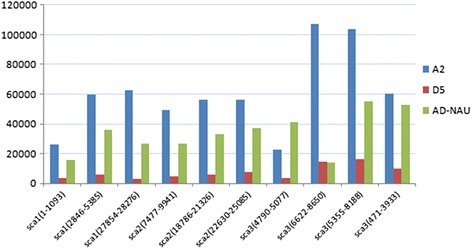
Copy number of the ten selected DNA fragments in A_2_, D_5_, and AD_1_ (Zhang et al. 2015 [36]) (hereafter we named it as AD_1_-NAU) genomes by BLASTN

By BLASTN using the BAC sequence as query and A_2_ draft genome (*G. arboretum*) [34] as subject sequence, we obtained ten DNA fragments (named after its sequence location in corresponding scaffold) from the BAC sequence, based on the more-than-80 % similarity and zero or approximate zero e-value. With the ten selected DNA fragments as query sequences, BLASTN were performed against D_5_ (*G. raimondii*) and AD_1_ (*G. hirsutum*) draft genomes [33, 36] respectively. After comparing the distribution of the ten fragments in different cotton genomes, it was found that the copy number was the highest in A_2_ genome, but 10–25 times lower in D_5_ genome (Fig. 2), and with very bad match hits (data not shown), which maybe partially explain the FISH results in D genome species. We extracted the sequences of the ten fragments from the BAC sequence for the following analysis.

**Fig. 3:**
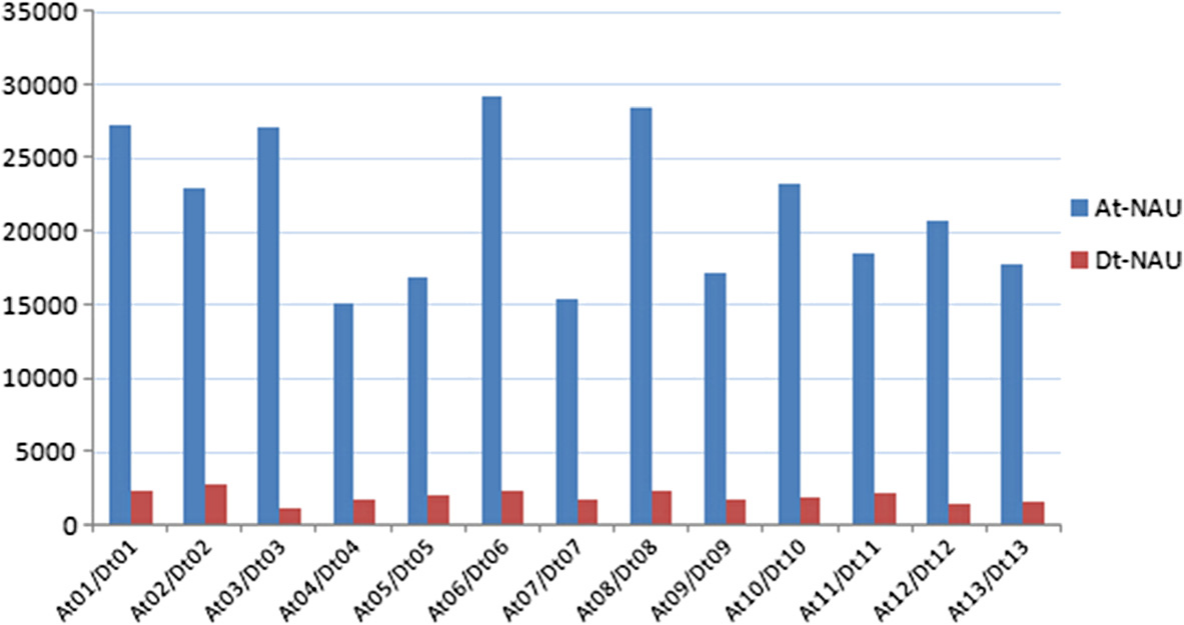
Total copy number of the ten fragments in every chromosome of AD_1_-NAU genome (At/Dt)

At the same time, taking into account the FISH results of BAC-57I23 in AD genome species, we compared the total repeated numbers of ten fragments in every chromosome of AD genome (Fig. 3). Result showed that the A sub-genome chromosomes had more than 10 times of repeats copy numbers than D sub-genome, and better consistency with the FISH results was viewed.

### Identification of repetitive sequences

**Fig. 4:**
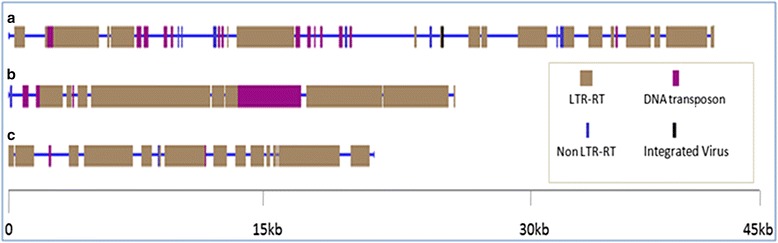
Sequence analysis graphical map of the repeat-rich bacterial artificial chromosome (BAC) clone 57I23. *Horizontal blue bars* represent the BAC sequence, *vertical bars* represent different repeat elements. *a*, scaffold1-42338 bp; *b*, scaffold2-26803 bp; *c*, scaffold3-22097 bp

Based on CENSOR results, DNA transposon, LTR-RT, Non-LTR-RT, and other repetitive elements were identified from the BAC sequences, which account for more than 62 % of the assembled BAC sequence. Among them, LTR-RT was predominant, accounting for 88.11 % of total identified repetitive elements (55.21 to 62.66 %) (Fig. 4 and Table 1). The identified LTR-RTs were classified into LTR/Gypsy, LTR/Copia, LTR/BEL families. Especially, LTR/Gypsy accounted for more than 91 % of the total identified LTR-RTs. By combining the CENSOR with BLASTN analysis results, we selected 12 LTR-RTs with higher score value (Table 2), and extracted the corresponding sequences from the BAC sequences for FISH verification.

**Table 1:** Summary of identified repeats in BAC sequence by CENSOR

Repeat class	Fragments	Length (bp)	Percentage accounting for BAC sequence
Integrated Virus	1	86	0.09 %
DNA transposon	25	6086	6.67 %
LTR Retrotransposon	94	50373	55.21 %
BEL	1	65	0.07 %
Copia	4	4251	4.66 %
Gypsy	89	46057	50.48 %
Non-LTR Retrotransposon	9	627	0.69 %
Total	129	57172	62.66 %

**Table 2:** Selected LTR-RTs from CENSOR results

Name	From	To	Name	From	To	Class	Name
scaffold1|size42338	4200	5326	Gypsy-48_GR-I	1376	2498	LTR/Gypsy	sca1 (4200-5326)
scaffold1|size42338	13782	17200	Copia-80_ST-I	194	3604	LTR/Copia	sca1 (13782-17200)
scaffold1|size42338	31153	32254	Gypsy-48_GR-I	3205	4308	LTR/Gypsy	sca1 (31153-32254)
scaffold2|size26803	7498	8637	Gypsy-48_GR-I	76	1221	LTR/Gypsy	sca2 (7498-8637)
scaffold2|size26803	23904	25399	Gypsy-18_GR-I	1326	2899	LTR/Gypsy	sca2 (23904-25399)
scaffold3|size22097	10623	11420	Gypsy-18_GR-I	2893	3697	LTR/Gypsy	sca3 (10623-11420)
scaffold3|size22097	17834	19556	Gypsy-48_GR-I	2773	4562	LTR/Gypsy	sca3 (17834-19556)
scaffold3|size22097	20731	21832	Gypsy-48_GR-I	3260	4363	LTR/Gypsy	sca3 (20731-21832)
scaffold2|size26803	13047	13565	Copia-2_JC-I	4861	5382	LTR/Copia	sca2 (13047-13565)
scaffold2|size26803	19133	19572	Gypsy-1_JC-I	276	715	LTR/Gypsy	sca2 (19133-19572)
scaffold2|size26803	18785	21330	Gypsy-48_GR-I	50	2596	LTR/Gypsy	sca2 (18785-21330)
scaffold3|size22907	5533	6040	Gypsy-1_JC-I	269	774	LTR/Gypsy	sca3 (5533-6040)

When running LTR-FINDER (version 1.05) using BAC sequence as query sequence, a 4118 bp full-length LTR-RT was identified in sequence region of scaffold1 (13558-17675). It belonged to the LTR/Copia family, and overlapped with Copia-80_ST-I identified by CENSOR.

By RepeatMasker (RepeatMasker vesion open-4.0.5) analysis, a 659 bp (sca2 (20662-21331)) Gypsy/DIRSI LTR element was identified, which had overlap region with sca2 (18785-21330) from the CENSOR results.

For further FISH verification, the partial above-mentioned fragments and LTR-RTs were PCR amplified and purified. Each purified DNA fragment had single band and expected size, which suited for the following work.

### Distribution of LTR-RTs in the cotton genomes

**Fig. 5:**
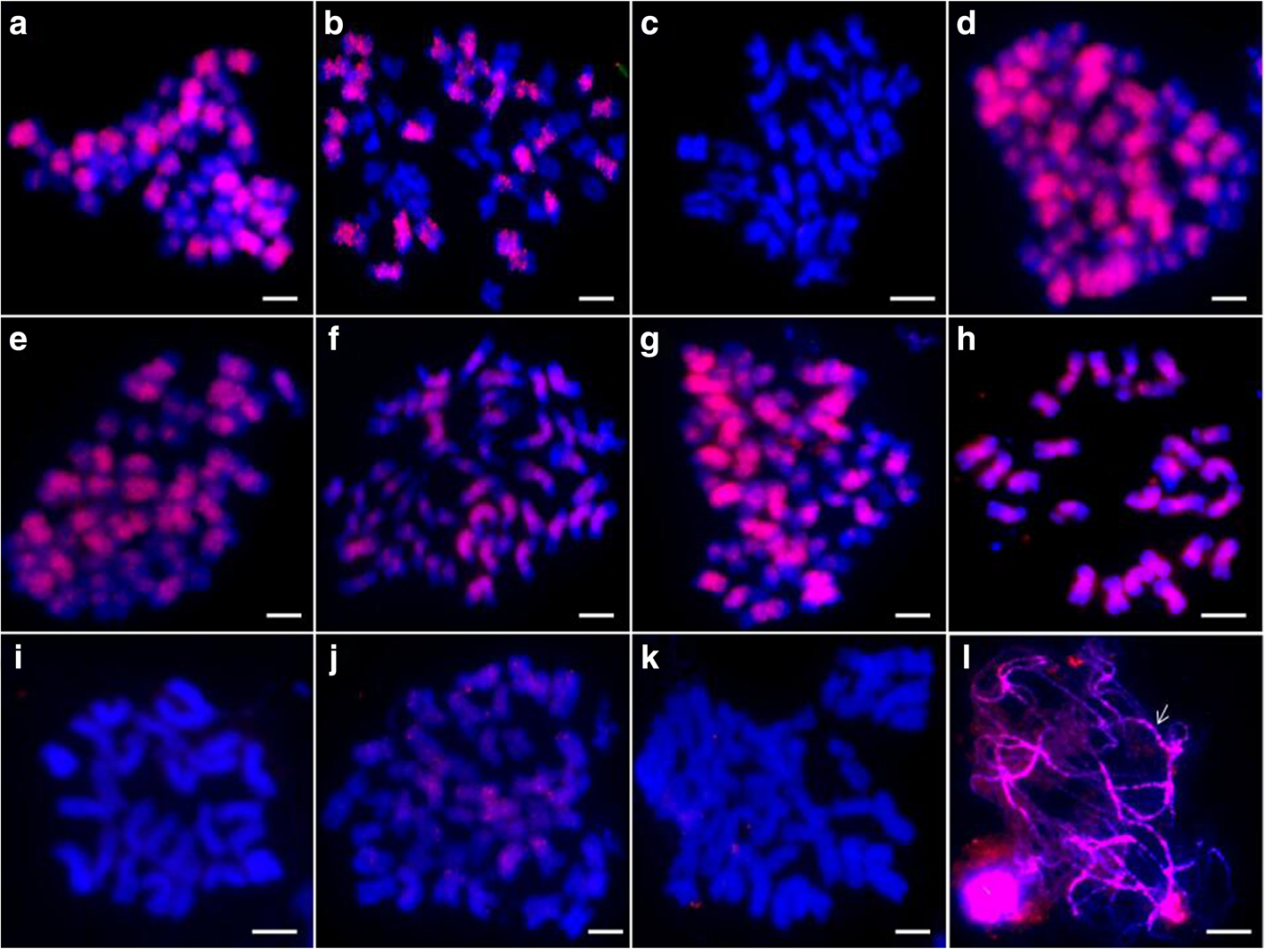
FISH analysis of distribution of identified LTR-RTs in cotton genome. **a**, sca2 (18785-21330)- *G. hirsutum*; **b**, sca3 (5355-8188)- *G. hirsutum*; **c**, sca3 (5355-8188)- *G. raimondii*.; **d**, sca1 (4200-5326)- *G. hirsutum*; **e**, sca2 (7498-8637)- *G. hirsutum*; **f**, sca3 (17834-19556)- *G. hirsutum*; **g**, sca3 (20731-21832)- *G. hirsutum*; **h**, sca1 (4200-5326)- *G. arboreum*; **i**, sca1 (4200-5326)- *G. raimondii*; **j**, scaffold1 (13558-17675)- *G. hirsutum*; **k**, sca2 (23904-25399)- *G. hirsutum*; **l**, sca2 (18785-21330)- *G. hirsutum* (pachytene). Bar = 5 μm

The FISH analysis of somatic metaphase chromosomes showed differential distribution patterns for each LTR-RT subfamily. When using Gypsy-48_GR-I-like LTR-RTs as probes, BAC-57I23-like signals were reappeared (Fig. 5a, d-i). Using sca3 (5355-8188) as probes, the FISH signals only were observed on chromosomes of A sub-genome with lower coverage relative to BAC 57I23 (Fig. 5b), and no signal on *G. raimondii* chromosomes (Fig. 5c). Using sca1 (13558-17675), a 4118 bp-LTR/Copia element as probe, only a few dotty signals appeared (Fig. 5j). But using sca2 (23904-25399), a Non-Gypsy-48_GR-I-like LTR-RT as probe, no signal appeared (Fig. 5k).

Pachytene chromosomes can display a differentiated pattern of heterochromatic and euchromatic regions [46, 49]. The pachytene-FISH results of *G. hirsutum* using fragment sca2 (18785-21330) as probe, which belonging to Gypsy-48_GR-I-like LTR-RT, showed high signal density throughout the partial pachytene chromosomes mainly following the distribution of heterochromatin, as white arrow shown (Fig. 5l).

## Discussion

### Sub-genome-specific cytogenetic marker

In early times, cotton chromosome identification was mainly based on the analysis of cytological characters, such as chromosomal relative lengths, arm ratios, and nuclear organization regions (NORs) in the mitotic or meiotic metaphase [50]. Because of the big number and small size of the chromosomes in cotton, the cytological identification of the chromosome has been hitherto limited. With the development of FISH, chromosome-specific FISH markers are effective tools for chromosome identification, analysis of genetic stocks, and physical mapping [13, 51–53]. BAC-57I23 displayed here can be used as a sub-genome specific FISH marker to identify A and D sub-genomes simultaneously in AD genome cotton species or allohexaploids containing A and D sub-genomes, due to the different FISH signal patterns on A and D sub-genome chromosomes. The discovery of BAC-57I23 provided a new FISH marker for identification of two or three sub-genomes at the same time, so the one-BAC FISH with 57I23 can take the place of GISH (genomic in situ hybridization) with two or three genomes DNA to achieve the identification of the sub-genomes.

### Assembly quality of repetitive sequences in allotetraploidy cotton draft genome

Decoding cotton genomes is a foundation for understanding the functional and agronomic significance of polyploidy and genome size variation within the *Gossypium* genus. But high-quality assembly of allopolyploid plant genomes is a formidable task because of the large genomes and the existence of highly homeologous sub-genomes [36]. Mis-assemblies are common when draft genome sequences have been generated by de novo assembly of sequences obtained with NGS technologies [54]. It’s possible that regions with repeated sequences might not be assembled successfully. FISH, allowing directly mapping of DNA sequences on chromosomes, has become an important technique in plant molecular cytogenetic research and can be used to guide draft genome assembly [37, 55, 56]. In this study, when blasting against the AD_1_-NAU draft genome using the identified repeats, the results had good consistency with the BAC-FISH results (Figs. 1 and 3). Based on this result, we can infer the assembly of the identified repetitive sequences in AD_1_-NAU draft genome has better matchup on their chromosome belonging.

### Genome size expansion and LTR-RTs

In diploid cottons, the A genome (1697 Mb) has nearly twice the size of the D genome (885 Mb) [1, 5]. The sequences analysis of cotton draft genome indicates that the amount of sequence encompassing LTR-type retrotransposons increased from 348 Mb in *G. raimondii* to 1145 Mb in *G. arboreum*, whereas the protein-coding capacities of these two species remained largely unchanged [32, 34]. In this study, the significant difference of FISH signal patterns of the BAC-57I23 between A and D genomes indicated that the BAC-57I23 should have specific composition, which can partly explain the size gap between A and D genome (Fig. 1g, h). By sequence analysis, a type of Gypsy-like LTR-RTs was identified as the key element in the BAC. The genomic distribution of the identified Gypsy-LTR-RT was similar to the distribution of heterochromatin (Fig. 5l). The expansion of this type of Gypsy-LTR-RT in heterochromatic regions may be one of the major reasons for the size gap between A and D genome. Here we provided visualized evidence by FISH that the proliferation of a type of Gypsy-like LTR-RTs is one of the major reasons for genome size diversity between A and D, which further supported the former studies results [8, 57, 58].

### The colonization of the genome

The previous studies showed that A-genome-specific dispersed repetitive sequences at the diploid level have colonized the D-genome at the polyploid level [38, 59]. Similarly, another study showed that a family of copia-like retrotransposable elements “horizontally” transferred across genomes following allopolyploid formation [60]. Page et al. discovered that approximately 900 kp of sequence in the polyploid genome have been converted from one genome to another in separate conversion events scattered across the genome by whole-genome re-sequencing [61]. Here, our results combined BAC-sequencing with FISH verification showed that a type of Gypsy-like LTR-RTs had high copies in *G. arboreum* (A_2_) genome, but none in the *G. raimondii*. (D5) genome (Fig. 5), however at the polyploidy level, obviously sequence expansion and colonization from A to D sub-genomes occurred, which dispersed on all D sub-genome chromosomes middle areas.

## Conclusions

As an excellent system for studying genome evolution and polyploidization, cotton cytogenetic study is increasingly on the agenda. Combined sequences analysis with FISH verification, a new genome-specific cytogenetic marker for identification of sub-genome was discovered. The repetitive sequences assembly quality of the allotetraploidy cotton draft genome was verified preliminarily, that is, the chromosome belonging of the repeats in AD_1_ draft genome has good consistency with the BAC-FISH results. A type of Gypsy-like LTR-RTs identified from the BAC-57I23 can partially explain the size gap between A and D genome. During the process of polyploidization of cotton, “horizontally” transferred from the A sub-genome to D sub-genome The findings showed here will help to understand the composition, structure, and evolution of cotton genome, and also will contribute to the further perfection of the draft genomes of cotton, as well as provided the cytogenetic evidence for polyploidy formation.
